# Surgical treatment and neurological outcome of infiltrating intramedullary astrocytoma WHO II–IV: a multicenter retrospective case series

**DOI:** 10.1007/s11060-020-03647-w

**Published:** 2020-10-22

**Authors:** Vicki M. Butenschoen, Vanessa Hubertus, Insa K. Janssen, Julia Onken, Christoph Wipplinger, Klaus C. Mende, Sven O. Eicker, Victoria Kehl, Claudius Thomé, Peter Vajkoczy, Karl Schaller, Jens Gempt, Bernhard Meyer, Maria Wostrack

**Affiliations:** 1 Department of Neurosurgery, School of Medicine, Klinikum Rechts Der Isar, Technical University Munich, Ismaningerstr. 22, 81675 Munich, Germany; 2 Department of Neurosurgery, University of Geneva, Rue Gabrielle-Perret-Gentil 4, 1205 Geneva, Switzerland; 3 Department of Neurosurgery, University of Innsbruck, Anichstr. 35, 6020 Innsbruck, Austria; 4 Department of Neurosurgery, University Hospital Hamburg Eppendorf, Martinistraße 52, 20251 Hamburg, Germany; 5 Department of Neurosurgery, Charité-Universitätsmedizin Berlin, Augustenburger Platz 1, 13353 Berlin, Germany; 6 School of Medicine, Institute of Medical Informatics, Statistics and Epidemiology, Technical University Munich, Grillparzerstr. 18, 81675 Munich, Germany

**Keywords:** Spinal astrocytoma, Intramedullary tumor, Spinal glioma

## Abstract

**Introduction:**

Primary malignant spinal astrocytomas present rare oncological entities with limited median survival and rapid neurological deterioration. Evidence on surgical therapy, adjuvant treatment, and neurological outcome is sparse. We aim to describe the treatment algorithm and clinical features on patients with infiltrating intramedullary astrocytomas graded WHO II–IV.

**Methods:**

The following is a multicentered retrospective study of patients treated for spinal malignant glioma WHO II–IV in five high-volume neurosurgical departments from 2008 to 2019. Pilocytic astrocytomas were excluded. We assessed data on surgical technique, perioperative neurological status, adjuvant oncological therapy, and clinical outcome.

**Results:**

40 patients were included (diffuse astrocytoma WHO II *n* = 11, anaplastic astrocytoma WHO III *n* = 12, WHO IV *n* = 17). Only 40% were functionally independent before surgery, most patients presented with moderate disability (47.5%). Most patients underwent a biopsy (*n* = 18, 45%) or subtotal tumor resection (*n* = 15, 37.5%), and 49% of the patients deteriorated after surgery. Patients with WHO III and IV tumors were treated with combined radiochemotherapy. Median overall survival (OS) was 46.5 months in WHO II, 25.7 months in WHO III, and 7.4 months in WHO IV astrocytomas. Preoperative clinical status and WHO significantly influenced the OS, and the extent of resection did not.

**Conclusion:**

Infiltrating intramedullary astrocytomas WHO II–IV present rare entities with dismal prognosis. Due to the high incidence of surgery-related neurological impairment, the aim of the surgical approach should be limited to obtaining the histological tissue via a biopsy or, tumor debulking in cases with rapidly progressive severe preoperative deficits.

## Introduction

Primary intramedullary spinal cord astrocytomas present rare case entities and account for 30–40% of all spinal cord gliomas [1–3]. Despite technical advances, the outcome of infiltrating astrocytomas remains poor [4–6], and patients suffer from rapid neurological deterioration [3, 5, 7]. The gold standard treatment remains a matter of debate as the extent of resection (EOR) does not necessarily correlate with tumor recurrence and may lead to clinical worsening of the patient [4, 5, 8]. While most published studies favor complete tumor resection [9], others question the therapeutic advantage of surgical resection [10–12]. Due to its rareness, data on adjuvant therapy such as combined chemo- and radiotherapy are sparse [13–15], and decisions on a patient’s optimal treatment are taken individually without defined guidelines. While most intramedullary astrocytomas of the spinal cord are low-grade tumors (World Health Organization WHO I or II tumors) [1, 16], studies on spinal high-grade gliomas are missing. In our study, we therefore analyzed the surgical approach and clinical outcome of patients suffering from infiltrating astrocytomas WHO II, III, and IV in order to better understand the influence of the surgical approach and adjuvant treatment on the overall survival (OS) and tumor recurrence.

## Methods

### Study cohort

We conducted a retrospective analysis of consecutive patients treated surgically for infiltrating primary intramedullary astrocytomas graded WHO II to WHO IV between January 2008 and July 2019. Tumor histology was defined according to the current WHO classification, before and after 2016. We excluded patients with pilocytic astrocytomas from the analysis due to the non-infiltrating nature and distinct clinical course of these tumors.

Then, we assessed motor function according to the Medical Research Council (MRC) scale. We used the modified McCormick Scale to assess the grade of the disability before and after surgery, as well as in the last follow-up examination.

Preoperative diagnostics included a contrast-enhanced magnetic resonance imaging (MRI) of the neuroaxis (brain and whole spine imaging) and analysis of cerebrospinal fluid (CSF). Intraoperative data included the EOR, use of intraoperative neurophysiological monitoring (IONM), and levels operated upon. We classified histopathological subgroups corresponding to their diagnosed WHO grade and recorded molecular markers (Alpha-Thalassemia/Mental Retardation Syndrome ATRX, Microtubule-associated protein MAP2, O6-methylguanine-DNA methyl-transferase MGMT, Isocitrate dehydrogenase IDH-1) in case they were analyzed.

Postoperative clinical and radiological follow-up examinations (with MRI) were conducted every 3 months after surgery.

### Study design

The study was a retrospective multicentric analysis conducted in 5 neurosurgical high-volume centers in Germany (3), Austria (1), and Switzerland (1). We analyzed relevant details on the type of surgery performed, the neurosurgical approach, preoperative clinical state (modified McCormick grading), early and long-term postoperative neurological function, tumor size and histopathological results (including the WHO grading of the tumor, molecular markers such as Ki-67, MGMT, and IDH-1 mutation), the EOR (gross total resection [GTR] defined as a complete removal of contrast enhancing tumor, subtotal resection [STR] defined as a removal of more than 90% of the contrast enhancing tumor, tumor debulking, or biopsy), and time to tumor recurrence.

### Statistics

We performed statistical analyses using SPSS Statistics 26 (IBM, Chicago, IL). Then, we compared categorical data using the chi-square test or Fisher’s exact test as needed. We also compared mean values using the independent samples *t* test. We analyzed the association between potential factors and the transient and permanent postoperative impairments (follow-up data or discharge data for those with missing follow-ups) using ANOVA and linear regression modeling. In addition, we assumed the following factors to be potentially predictive: IONM, WHO of the tumor, EOR, adjuvant treatment, and age for univariate and multivariate analysis. To assess the correlation, we used Kendall’s Tau correlation coefficient. We assessed overall survival using the Kaplan–Meier estimator and compared the survival curves using the log-rank test. All tests were performed two sided at the 5% significance level.

### Ethical considerations

We executed the presented study in accordance with the ethical standards outlined in the Declaration of Helsinki. Also, we obtained a positive vote by a local ethics committee beforehand (Number 5766/13). Due to the retrospective nature of the study, prospective patient consent was not required.

## Results

### Patient population

In total, we included 40 patients for analysis. Median age was 41 years (range 4–85 years). Twenty-four patients were male (60%), and 16 were females (40%) (Table 1).

**Table 1:** Demographics, tumor localization (CTJ: cervicothoracic junction, TLJ: thoracolumbar junction, multiple: including cervical, thoracic and lumbar spine), first diagnosis and median preoperative McCormick grade

	WHO II	WHO III	WHO IV	Total	p
	n = 11	n = 12	n = 17	N = 40	
Median age in years (range)	40 (30–85)	41 (18–78)	36 (4–70)	41 (4–85)	0.224^§^
Female sex (%)	36.4	33.3	47.1	40	0.728*
Localization n (%)					0.832^#^
Cervical	2 (18)	2 (17)	2 (12)	6 (15)	
CTJ	1 (9)	2 (17)	5 (29)	8 (20)	
Thoracic	5 (45)	3 (25)	5 (29)	13(32.5)	
TLJ	2 (18)	4 (33)	2 (12)	8 (20)	
Lumbar	1 (9)	0 (0)	1 (6)	2 (5)	
Multiple	0 (0)	1 (8)	2 (12)	3 (7.5)	
Previous surgery n (%)	3 (27.3)	2 (16.7)	5 (29.4)	10 (25)	0.810^#^
Preoperative McCormick (median)	II	III	III	III	0.315*

P = level of significance

^$^Anova

*Chi^2^ Test

^#^Fishers exact test

Ten patients were diagnosed with an infiltrating astrocytoma of the spinal cord prior to the presentation at our departments surgically, without prior radio- or chemotherapy (25%).

The majority of the patients had already presented with moderate neurological disability (McCormick Grade III 19/40 patients, 47.5%) before surgery. Intact neurological function or mild impairment with functional independency was assessed in 40% (16/40) of the patients (McCormick Grade I in 5/40 [12.5%] and II in 11/40 [27.5%]). Tumor lesions were dominantly localized in the thoracic spinal cord (13/40, 32.5%), followed by the cervico-thoracic and thoraco-lumbar junction (each 8/40, 20%). Multiple lesions extending to the cervical, thoracic, and lumbar spine were detected in 3/40 patients (7.5%) (Table1). In 32/40 patients (80%), CSF diagnosis was performed before surgery. Pathological cells were identified in only 6/32 patients (18.7%).

Mean duration of symptoms was 179 days (range 1 day to 3 years). In a subgroup analysis, the mean duration of symptoms was 301 days in WHO II tumor patients, 220 days in WHO III tumor patients, and 84 days in patients with a WHO IV tumor. The differing mean values showed a trend of dependence on the WHO grade without reaching statistical significance (*p* = 0.093) in ANOVA modeling.

### Surgical approach

IONM with motor-evoked potentials (MEPs) and somatosensory-evoked potentials (SSEPs) was conducted in 58.3% of the surgeries performed. All surgeries were performed in the prone position. Eighteen patients underwent a biopsy (18/40, 45%), followed by STR (15/40, 37.5%) and tumor debulking (5/40, 12.5%), and only 2/40 patients underwent a GTR (5%).

Regarding the surgical approach, we chose the following approaches (in decreasing order): laminectomy (LE, 16/40 patients, 40%), hemilaminectomy (HL, 10/40 patients, 25%), laminoplasty (LP, 9/40 patients, 22.5%), decompression and dorsal fixation (Fix, 3/40 patients, 7.5%), and interlaminar fenestration (ILF, 5%, only for biopsies in 2 patients).

The mean number of segments operated upon was 3.65 ± 2.9 (range 1–14), without any significant difference between subgroups (*p* = 0.687).

### Outcome

#### Histopathology

Of the patients, 11/40 were diagnosed with WHO II diffuse astrocytomas of the spinal cord (27.5%). In 12/40 patients, the histopathological analysis revealed an anaplastic astrocytoma WHO III (30%), and 17/40 patients suffered from a glioblastoma or diffuse midline glioma (both graded WHO IV, 42.5%).

The Ki-67 expression index was assessed in 32/40 patients (80%). Median Ki-67 expression was 17.5% (range 0 to 50%, interquartile range 5–20%), which is significantly dependent on the WHO grade of the operated tumor (median Ki-67 expression in WHO II tumors 3.5%, WHO III tumors 15%, WHO IV tumors 20%, *p* = 0.006). Table 2 summarizes other molecular markers (IDH-1, MGMT, ATRX, MAP). Loss of nuclear ATRX expression showed a strong association with the WHO (*η*^*2*^ = 0.481) but failed to reach statistical significance (*p* = 0.052) (Table 2).

**Table 2:** Number of patients analyzed (n) and analysis of tumor positivity for MAP12 (Microtubule-associated Protein-2), IDH1 (Isocitrat Dehydrogenase 1), H3K27M (Methylation of Histone H3 on Lysine 27), MGMT (Methylguanin-Methyltransferase), loss of ATRX expression (alpha thalassemia/mental retardation syndrome X-linked, p = 0.052) and Ki-67 (*significant difference p < 0.01), η^2^ describes the effect size

Marker	WHO II	WHO III	WHO IV	p	η^2^
MAP2				0.078	0.259
Tested (n)	4	7	9		
Positive (%)	50	100	89		
IDH1				0.121	0.168
Tested (n)	5	8	13		
Mutated (%)	20	0	0		
H3K27M				0.178	0.400
Tested (n)	0	1	5		
Mutated (%)	0	0	80		
MGMT				0.269	0.213
Tested (n)	1	5	8		
Methylated (%)	100	60	25		
ATRX				0.052	0.481
Tested (n)	2	3	7		
Loss of expression (%)	50	67	0		
Ki67				0.006*	0.301
Tested (n)	8	11	13		
Median (%)	3.5	15	20		

#### Clinical outcome

We observed new neurological deficits in 49% of the cases postoperatively. After surgery, only 25.6% of the patients were classified as functionally independent (McCormick Grade I or II with no or only mild neurological impairment), and the majority presented with moderate neurological impairment (McCormick Grade III, 46.2%) and severe impairment (McCormick Grade IV, 20.4%). Three patients suffered from paraplegia, classified as Grade V on the modified McCormick grading scale (7.7%).

Regarding the clinical outcome at discharge compared to the preoperative neurological status, 46.2% of the patients kept a stable neurological impairment. Also, 38.5% suffered from a deterioration of the neurological function, and 7.7% had an improved function. Thirty-day mortality was 7.7% (*n* = 3), with death being caused by respiratory distress (*n* = 2) and pulmonal artery embolism (*n* = 1). None of the patients died of surgery-related complications and all of them presented with a preoperative McCormick Grade III.

We did not find any significant association between the number of segments operated upon (p = 0.232), the approach (*p* = 0.082), IONM (*p* = 0.073), or the EOR (*p* = 0.411) on postoperative neurological outcome. The WHO of the tumor was associated with the postoperative McCormick grade in univariate analysis (*p* = 0.02).

#### Adjuvant treatment, tumor recurrence, overall survival, and follow-up

Decisions about adjuvant therapy were met within the interdisciplinary tumor board for each patient. Patients with diffuse astrocytoma WHO II were either assigned to a “wait and see” algorithm (*n* = 4, 36.4%), underwent fractioned radiotherapy (*n* = 3, 27.3%, total dose 50.4 Gy, dose per fraction 1.8 Gy), or were treated with combined fractioned radio- and chemotherapy (*n* = 4, 36.4%) (with Temozolomide TMZ 75 mg to 150 mg per body surface area per day in all cases following the Stupp protocol). Decision on the postoperative treatment did not depend on the EOR (*p* = 0.136). Patients with anaplastic intramedullary astrocytoma WHO III received combined chemo- and radiotherapy in 83% of cases (10/12, fractioned radiotherapy with concomitant TMZ). Patients with glioma WHO IV were treated with adjuvant combined Stupp protocol therapy in 16 cases (94%). One patient underwent only chemotherapy (TMZ) due to his limited clinical postoperative status. Two patients with WHO III and 1 patient with a glioblastoma died before finishing their adjuvant treatment within 30 days.

Median time to follow-up was 299 days (9.8 months, range 3 days: 2,500 days). Three patients had died, and 2 patients were lost to follow-up.

McCormick grade at follow-up had deteriorated to severe neurological impairment in 10 patients (McCormick Grade IV in 27.8%). Also, 30.6% still had a moderate impairment (McCormick Grade III), and 22.2% (*n* = 8) already suffered from paraplegia or tetraplegia (McCormick Grade V). Only 19.4% (*n* = 7) remained functionally independent (McCormick Grade I or II).

**Fig. 1:**
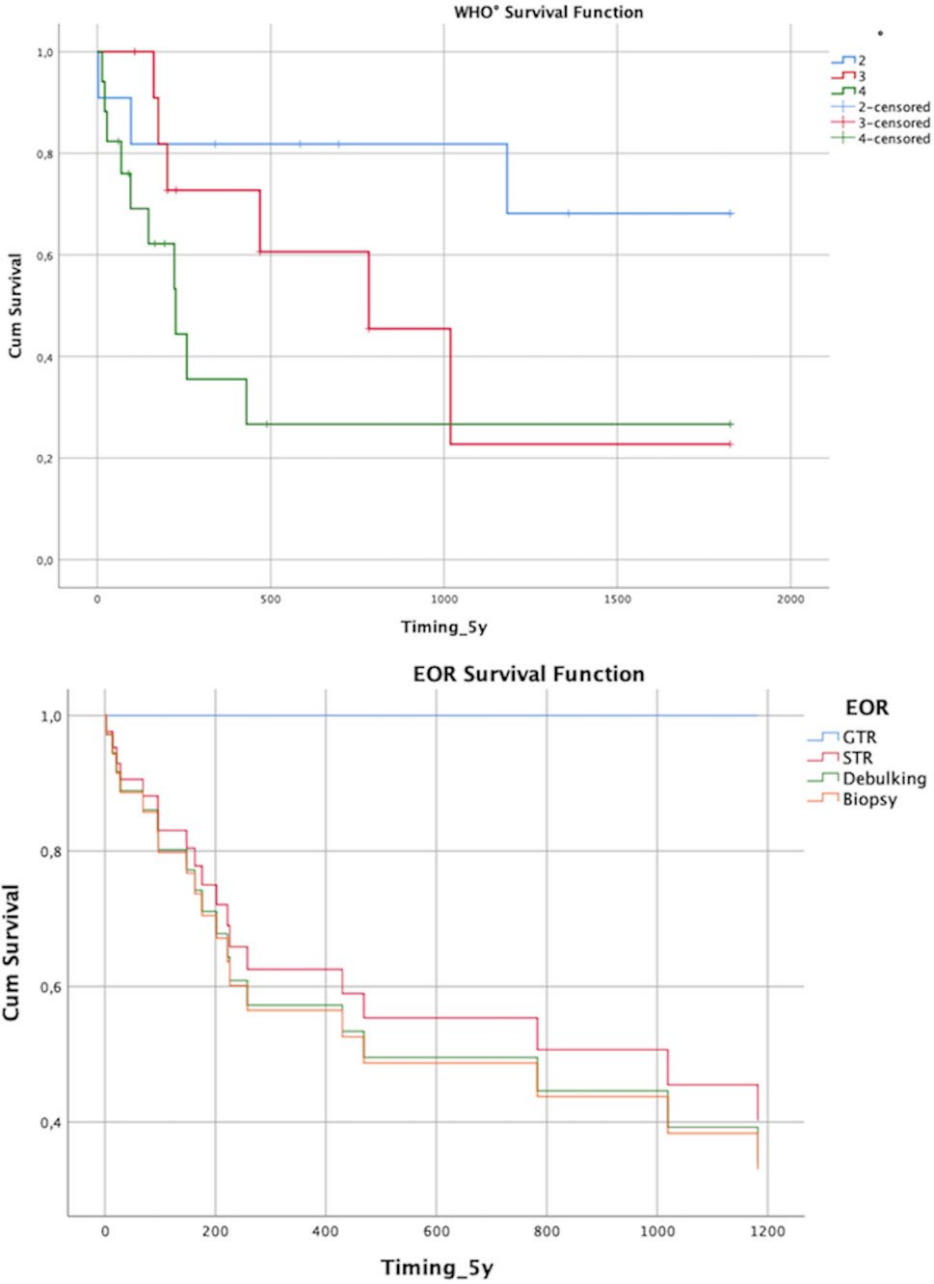
Overall survival depends on tumor histology and WHO grade (II-IV) with a steep drop in survival probability in patients with WHO IV tumors and a flatter line in patients with WHO II tumors (upper image, *p* = 0.052); Survival function in relation to the extent of resection (EOR) categorized in tumor biopsy, tumor debulking, subtotal tumor resection (STR), and gross total resection (GTR) (lower image, *p* = 0.616)

**Fig. 2:**
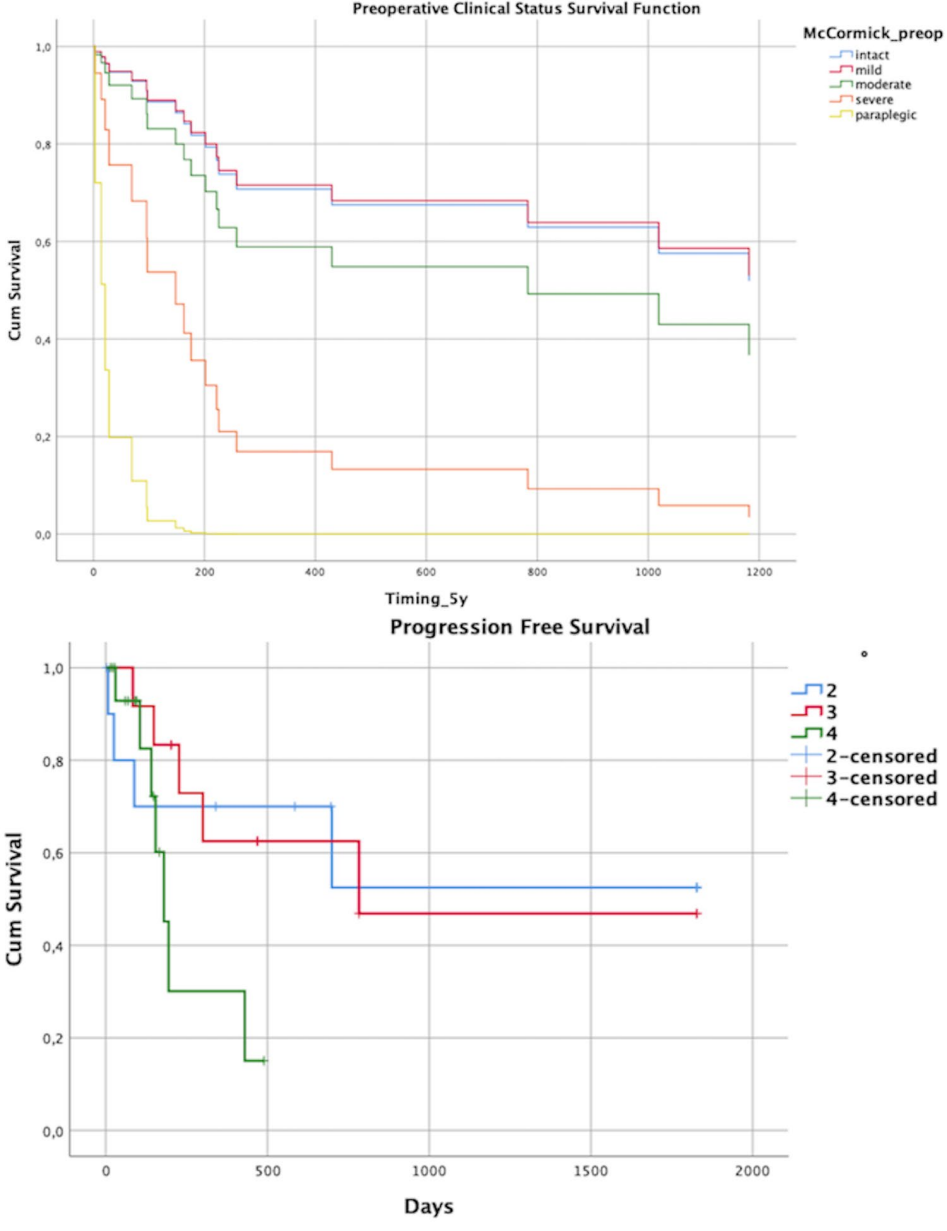
Survival function depends on the preoperative McCormick grade, divided into intact neurological status (I), mild symptoms (II), moderate impairment (III), severe neurological dysfunction (IV), and paraplegia (V) (upper image, *p* = 0.005); Progression-free survival (PFS) in relation to the WHO grade of the tumor (lower image, *p* = 0.154)

**Fig. 3:**
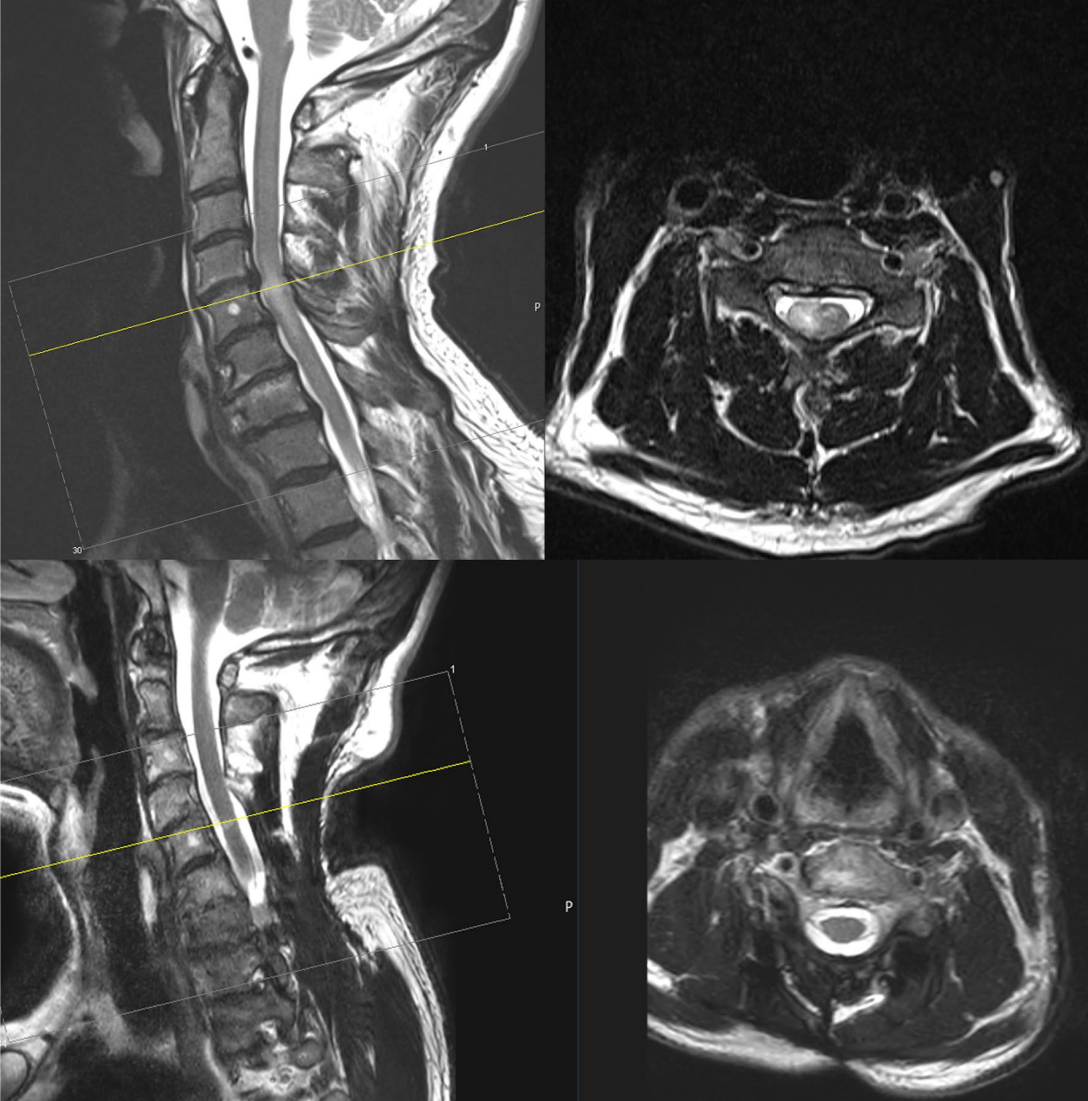
Preoperative (upper image) T2 sequenced MRI of a patient suffering from an intramedullary diffuse astrocytoma WHO II C4–6 with complete tumor resection and MRI imaging after 7 years (lower image) without tumor recurrence

We point censored the survival data to 5 years. Median overall survival was dependent on the WHO grade of the tumor (*p* = 0.052, log-rank test). WHO II tumor patients had a median survival of 46.5 months, WHO III patients had a median survival of 25.7 months, and WHO IV patients had a median survival of 7.4 months after surgery (Table 3, Fig. 1). The EOR had no significant effect on median survival (*p* = 0.616, Fig. 2), whereas the preoperative modified McCormick score significantly influenced the survival course (*p* = 0.005, Fig. 3), and the postoperative McCormick score did not (*p* = 0.487).

**Table 3:** Estimates from Kaplan–Meier curves for median survival of patients with WHO II tumor (46.5 months), WHO III (25.7 months) and WHO IV (7.4 months) with standard (Std.) error and 95% confidence interval, censored for 5 years, p = 0.052 (Log-Rank Test)

WHO*	Median	95% confidence interval	
	Estimate (days)	Lower bound	Upper bound
II	1827	1131	2523
III	1019	547	1491
IV	226	107	345
Total	783	19	1547

Local recurrence or tumor progression occurred in a total of 16 patients (40%) (WHO II: 4, WHO III: 5, WHO IV: 7) after a median of 187 days, depending on tumor histology (*p* = 0.012, WHO II: 585 days, WHO III: 384 days, WHO IV: 106 days) (Fig. 2). Median progression-free survival (PFS) was 783 days for WHO III tumors and 180 days for WHO IV tumors (*p* = 0.154) (Fig. 2).

Center effects were accounted for by including the study center as a potential predictor in the model. There was no statistical correlation between the participating center and the EOR, PFS and postoperative McCormick score, respectively in the univariate linear regression models (p-values ≥ 0.634), rendering the data independent of the treating unit.

### Case presentation

#### Case 1

A 57-year-old patient presented with progressive myelopathic gait ataxia and symptoms at our outpatient department. Spinal MRI imaging showed a T2 hyperintense lesion at the level C4-C6 (Fig. 3) indicating an intramedullary tumor (low grade intramedullary astrocytoma vs. ependymoma). The patient underwent a laminectomy C5 and gross total tumor resection. In the postoperative course, the patient complained of burdening dysesthesia of the legs and unchanged myelopathy symptoms. Histopathology revealed a diffuse astrocytoma WHO II, and the patient underwent adjuvant fractioned radiation therapy (total dose of 50.4 Gy over 6 weeks). 7 years later, the patient remains recurrence-free (Fig. 3).

#### Case 2

**Fig. 4:**
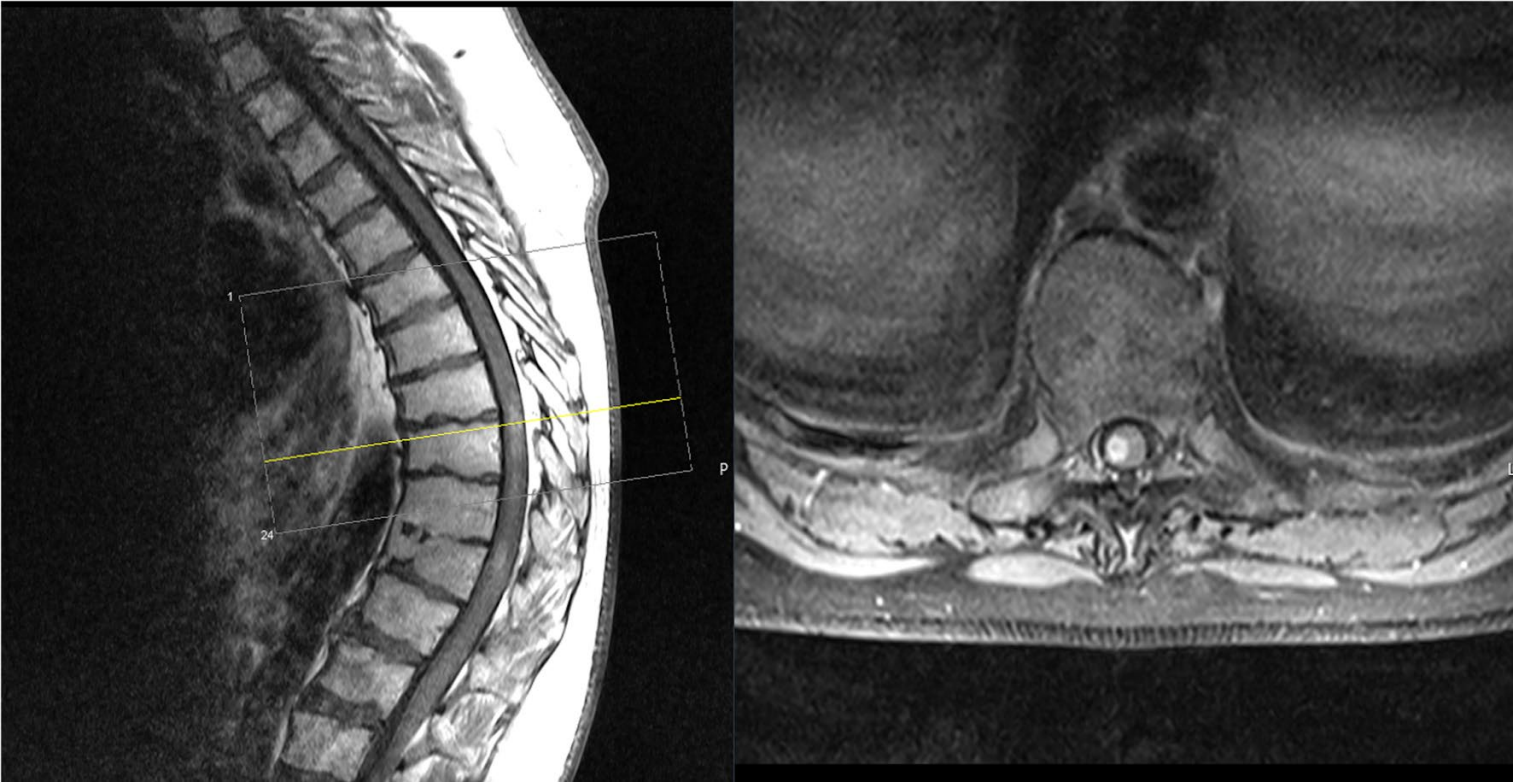
Preoperative MRI (T1 with contrast enhancement) of a patient suffering from an intramedullary Glioblastoma WHO IV at the level Th8-10

A 41-year-old patient presented with an incomplete paraparesis of the lower extremities with hypesthesia below Th10 and impaired micturition. Symptoms started a week ago, and MRI imaging revealed a contrast-enhancing intramedullary lesion from Th8 to Th10 (Fig. 4). A biopsy through a laminectomy Th9 was performed and revealed a Glioblastoma WHO IV (IDH-1 wild type, no MGMT methylation). After the biopsy, the patient showed further neurological deterioration with a complete paraplegia of the lower extremities. Patient was advised to undergo combined chemo- and radiotherapy (fractioned photon therapy, total dose of 50.4 Gy with concomitant 150 mg TMZ/day). The patient suffered from a fatal pulmonary artery embolism during the adjuvant therapy and died 4 weeks after surgery.

## Discussion

### Clinical outcome

The neurological outcome and deterioration after surgery did not significantly correlate with the tumor entity and WHO grading, whereas the follow-up data suggested a strong correlation between the WHO grade and overall survival as well as long-term functionality, similar to current evidence [5, 15, 17]. In a case series of 20 patients published in 2016, the authors reported a neurological deterioration in 51.5% of the patients postoperatively [18], which is consistent with our results (neurological deterioration in 48.8%). The risk for neurological deterioration has been associated with the EOR [19, 20], with clinical impairment after extensive tumor resection compared to minimal invasive tumor biopsy. At follow-up, more than 80% of our patients suffered from at least moderate neurological impairment (modified McCormick grade >  = III). Neurological impairment and worsening of clinical symptoms unfortunately correspond to the natural history of infiltrating spinal astrocytomas, and the progression of the neurological decline highly depends on the tumor entity (WHO ) [5, 8]. Median survival in patients with WHO IV tumors was only 7.4 months. Compared to the published literature, the median overall survival rates are 9 or 10 months [6, 21–24] to 15 months, including astrocytoma WHO III tumors [18] In our study, three patients died before completing adjuvant therapy. Interestingly, we found two patients in our cohort to have histologically proven WHO IV tumors and a long-term survival of more than 5 years. Case reports have been published describing long-term survival associated with cordectomies [25], which was not performed in any of our patients. We point censored the survival data to 5 years. Without censoring the data, survival was still significantly dependent on the WHO (*p* = 0.026 log rank test), therefore excluding a selection and timing bias.

### Prognostic factors for survival

Several studies have focused on the prognostic value of the EOR on overall survival and PFS in analogy to cranial astrocytomas. We did not observe any statistical association of surgical extent and survival, but, interestingly, we identified a highly significant prognostic clinical factor: the preoperative clinical status (classified via the modified McCormick grade) in univariate and multivariate testing. The influence of clinical factors has been described with ambiguous results [18, 26].

### Surgical treatment

The scope of surgery in patients with infiltrating astrocytomas WHO II-IV remains controversial. In our multicenter study, we found a heterogeneous population of surgical strategies including GTR, STR, debulking, and biopsy. A standard operative treatment has not yet been determined and has been discussed extensively [6, 27]. GTR or even STR in patients with infiltrating astrocytomas of the spinal cord is considerably difficult to achieve and requires to identify a surgical plane [5]. Congruent with the current literature, GTR was performed in a minority of the cases (6.8%) in our multicenter study. Furthermore, due to a high rate of postoperative neurological deterioration after (subtotal or gross-) tumor resection [18] the beneficial effect of extensive surgery on overall and progression-free survival in high-grade intramedullary tumors has been controversially discussed [4, 18, 28–30]. In some studies, surgical treatment showed a slight benefit without reaching any statistical significance [22], and others reported a negative prognostic effect of extensive tumor resection on survival and neurological status [20, 26]. In our study, OS did not significantly depend on the type of surgery performed (*p* = 0.79). Due to the limited number of cases, we may not detect a significant statistical influence, which is clearly a limitation of our study. However, due to an unacceptably high risk of postoperative impairment and doubtful benefit for survival, we would not advocate enforced extensive resection of infiltrating spinal cord glioma. Moreover, as observed in our study, overall survival seems to be significantly dependent on the patient’s functional status, rendering the surgical safety one of the most important prognostic factors.

In conclusion, the operative scope of surgery should be questioned and discussed. Superiority of GTR or STR in patients with high-grade intramedullary astrocytomas failed to be proven, and neurological deterioration and limited functionality should be taken into consideration while discussing optimal surgical treatment options with the patient.

### Adjuvant treatment

In total, 83% of the patients with intramedullary WHO III astrocytomas and 94% of the patients with WHO IV tumors underwent a combined chemo- and radiotherapy. We only made exceptions in cases of reduced clinical status or best supportive care decisions. The adopted standard treatment has been defined by studies including and analyzing patients with intracranial pathologies [31], as we lack randomized trials including patients with infiltrating spinal astrocytomas due to the rarity of the described tumor entity. Recommendations for adjuvant treatment are based on trends in improvement of OS and PFS rather than evidence-based trials [32] and derived from the analogy to the intracranial counterparts. In spinal cord astrocytomas, radiotherapy failed to reach a statistically significant effect in most publications [29, 33], although a positive trend has been described [22]. Chemotherapy with TMZ has been considered beneficial in patients with malignant spinal cord astrocytomas [16, 34], without reaching statistical significance (*p* = 0.57) [35]. Statistical analysis of all series, however, is limited by the small number of cases analyzed. In our study, we did not compare different adjuvant modalities due to the retrospective nature of our data and the fact that almost all patients with high-grade intramedullary astrocytomas underwent combined adjuvant treatment. A comparison between treatment modalities is therefore not possible.

### Molecular markers

Analyses for the presence of molecular markers such as H3K27M, MGMT, ATRX, and IDH-1 did not show any significant influence on the overall survival, although we only analyzed a small number of specimens. Ki-67 was significantly dependent on the WHO , and the assessed mean index was comparable to the published literature (mean Ki-67 index of 16.7%) [36]. Loss of ATRX expression showed a strong dependence but failed to reach statistical significance, possibly due to the small sample size of only 12 examined specimens out of 40. We did not detect any loss of ATRX expression in WHO IV gliomas, but in 2/3 of anaplastic gliomas, which is congruent with current literature [37]. Only a few studies have analyzed molecular markers in intramedullary astrocytomas but have described similar results [38]. Interestingly, IDH-1 could not be identified in any of the high-grade tumor specimens, which is congruent with the current literature [39], suggesting that glioblastoma of the spinal cord may correspond only to primary high-grade glioma. In a recent study investigating the genetic profile of intramedullary astrocytomas, Zhang et al. postulated that astrocytomas of the spinal cord may arise from alternative mechanisms of tumorigenesis compared to intracranial astrocytomas [40]. Due to their possibly distinct genetic profile and nonconformity with their intracranial counterparts, genetic alterations should be discussed for targeted therapy, regarding the limited prognosis of patients with spinal infiltrating astrocytomas with current treatment strategies [40–42]. Further molecular analysis is needed for evaluation of tumor origin and possible therapeutic agents [42], as neuropathological diagnostics evolve and may open further diagnostics paths for molecular characterization in small tumor samples.

### Description of the principal findings and conclusions drawn

Intramedullary infiltrating astrocytomas WHO II-IV present rare entities, with limited neurological outcome and dismal prognosis. Tumor entity (WHO grading) significantly influences the neurological outcome at discharge and median survival. Extent of tumor resection did not statistically influence the median survival and neurological deterioration that occurred in almost 50% of the cases. Interestingly, the preoperative McCormick grade significantly affected the overall survival, and molecular markers such as Ki-67 and ATRX show an effect on prognosis which has not been described in intramedullary gliomas before.

We recommend a surgical approach limited to obtaining the tumor histology (biopsy) or tumor debulking in patients with severe preoperative deficits. Standard adjuvant treatment with combined chemo- and radiotherapy should be performed in the majority of patients with high-grade intramedullary tumors, based on published recommendations rather than evidence.

## Data Availability

The datasets used and/or analyzed during the current study are available from the corresponding author on reasonable request.
